# Kawasaki Disease Shock Syndrome in the Eastern Region of Saudi Arabia: Case Series

**DOI:** 10.7759/cureus.14961

**Published:** 2021-05-11

**Authors:** Fadi Busaleh, Sajjad M AlKadhem, Aymen Albarrak, Abdullah A Almubarak, Mahmoud M Aldandan, Jumanah M Almajed, Mujtaba A Alabdullah, Luay F Almulaifi

**Affiliations:** 1 Pediatrics, Maternity and Children Hospital, Al-Ahsa, SAU; 2 College of Medicine, King Faisal University, Al-Ahsa, SAU; 3 Collage of Medicine, King Faisal University, Al-Ahsa, SAU

**Keywords:** kawasaki disease, kawasaki disease shock syndromes, shock, incomplete kawasaki disease, multi-system inflammatory disease in children (mis-c)

## Abstract

Kawasaki disease (KD) is a treatable medium-sized vasculitis in the pediatric population consisting of a myriad of specific signs and symptoms. A new entity of the disease, Kawasaki disease shock syndrome (KDSS), is defined as a KD patient presenting with signs of hypoperfusion. Our aim is to describe the signs and symptoms of KDSS and how it is treated and its consequences. Out of 37 patients diagnosed with KD in the period between January 2018 and December 2019 in hospitalized patients younger than 14 years of age at Maternity and Children's Hospital in Al-Hassa, Eastern Province, Saudi Arabia, 3 (8.10%) patients fulfilled the diagnostic criteria for KDSS: 2 (66%) were male and 1 (33%) was female. The cardinal feature in all of them was peripheral cardiovascular collapse. Two patients (66%) were found to have aseptic meningitis. All patients were treated with immunomodulatory agents (intravenous immunoglobulin) and all responded well to anti-inflammatory doses of aspirin. KDSS is the shock state of KD presenting with hypoperfusion symptoms, mainly irritability and changes in the level of consciousness and peripheral cardiovascular collapse. Awareness of such presentation and management by immunomodulatory medications helps in recovery and prevention of tragic consequences of such disease.

## Introduction

Kawasaki disease (KD) is an acute febrile multisystemic illness of pediatrics with an estimated incidence of 9 to 20 per 100,000 children under five years of age. Pathologically, it is characterized by inflammation of small to medium-sized extraparenchymal arteries [1]. Until 1967, KD was thought to be a subtype of mucocutaneous-ocular syndrome when Dr. Tomisaku Kawasaki reported fifty cases with a unique presentation. Since then, it has been identified as KD [2]. Although it has been suggested that the inflammatory process in KD is triggered by one or more unidentified infections, the etiology remains unknown in genetically susceptible individuals [1].

KD can be diagnosed by the persistent fever for at least five days with at least four signs of the following criteria: (I) bilateral non-purulent conjunctivitis sparing the limbus, (II) oropharyngeal mucosal changes including red cracked lips, strawberry-like tongue or diffuse erythematous oropharynx, (III) nonspecific generalized rash, (IV) swelling and erythema of the extremities, and (V) unilateral cervical lymphadenopathy. Incomplete KD is most commonly seen in infants and older children with fewer criteria and additional laboratory or echocardiographic findings suggestive of KD [1,2].

Early diagnosis of KD is necessary to reduce the risk of coronary artery lesions (CALs), which can be complicated by myocardial infarction and death [1]. Another rare complication is Kawasaki disease shock syndrome (KDSS), which was first reported by Kanegaye et al. [3]. KDSS is characterized by the presence of Kawasaki disease with either hypotension (20% drop in normal systolic pressure) or signs of hypoperfusion with increased risk of cardiovascular complications and extreme abnormality of inflammatory markers [3]. KDSS is poorly represented in the Kingdom and worldwide, although it shares numerous similarities with multisystemic inflammatory shock in children with COVID-19 infection but is poorly described. Therefore, we aimed to enrich the literature with information on the presentation, behavior and management of KDSS.

## Case presentation

Case 1

**Figure 1:**
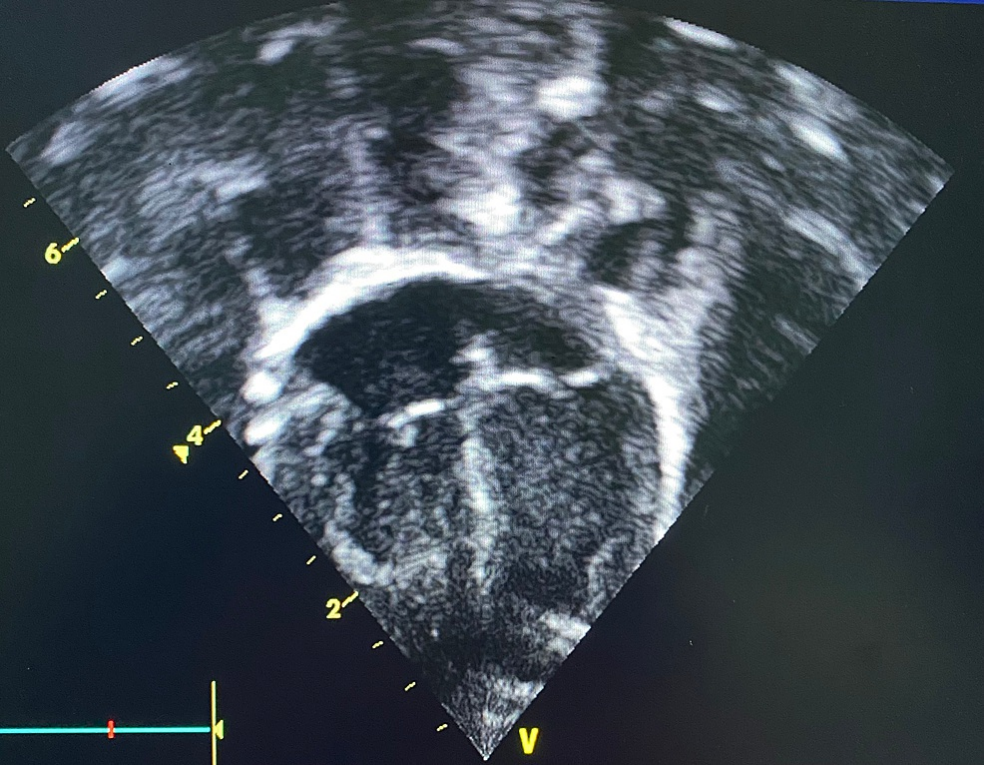
Echocardiogram, four-chamber view with normal findings without cardiac abnormality or fluid accumulation.

A 14-month-old Saudi girl, with no known medical illness, presented with the chief complaint of unremitting fever and jaundice for four days. Other major findings were vomiting, mucocutaneous involvement on the face, bilateral non-purulent conjunctivitis, angular stomatitis, chapped lip and generalized maculopapular rash on body and perianal desquamation. Her vital signs were conspicuous for fever (38.4 degree C), tachycardia (177 pulses/min), a steady blood pressure (85/50 mmHg), but a delayed capillary refill time (more than 4 seconds). Initial laboratory tests were significant for pancytopenia, direct hyperbilirubinemia, elevated liver enzymes, coagulopathy, and inflammatory markers. The patient was initially admitted to the pediatric intensive care unit (PICU) as a case of compensated septic shock, where a septic workup was performed and he was started on broad-spectrum antibiotics (cefotaxime and vancomycin) with rescues normal saline boluses (60 ml/kg over 1 hour), with coagulopathy corrected by fresh frozen plasma (FFP) and vitamin K. Prefusion improved by inotropic support. Abdominal ultrasound was unremarkable, while echocardiogram was normal (Figure 1). Because symptoms of atypical Kawasaki syndrome and signs of hypoperfusion were present, a diagnosis of Kawasaki shock syndrome was made on the second day of admission. The patient was treated with intravenous immunoglobulin (IVIG) 2 g/kg and aspirin 80 mg/kg/day (anti-inflammatory dose ), distributed every six hours. On the fifth day, the fever resolved completely and the patient was switched to the single antiplatelet dose of aspirin (5 mg/kg/day) and discharged home, with follow-up by pediatric cardiology in six weeks.

Case 2

**Figure 2:**
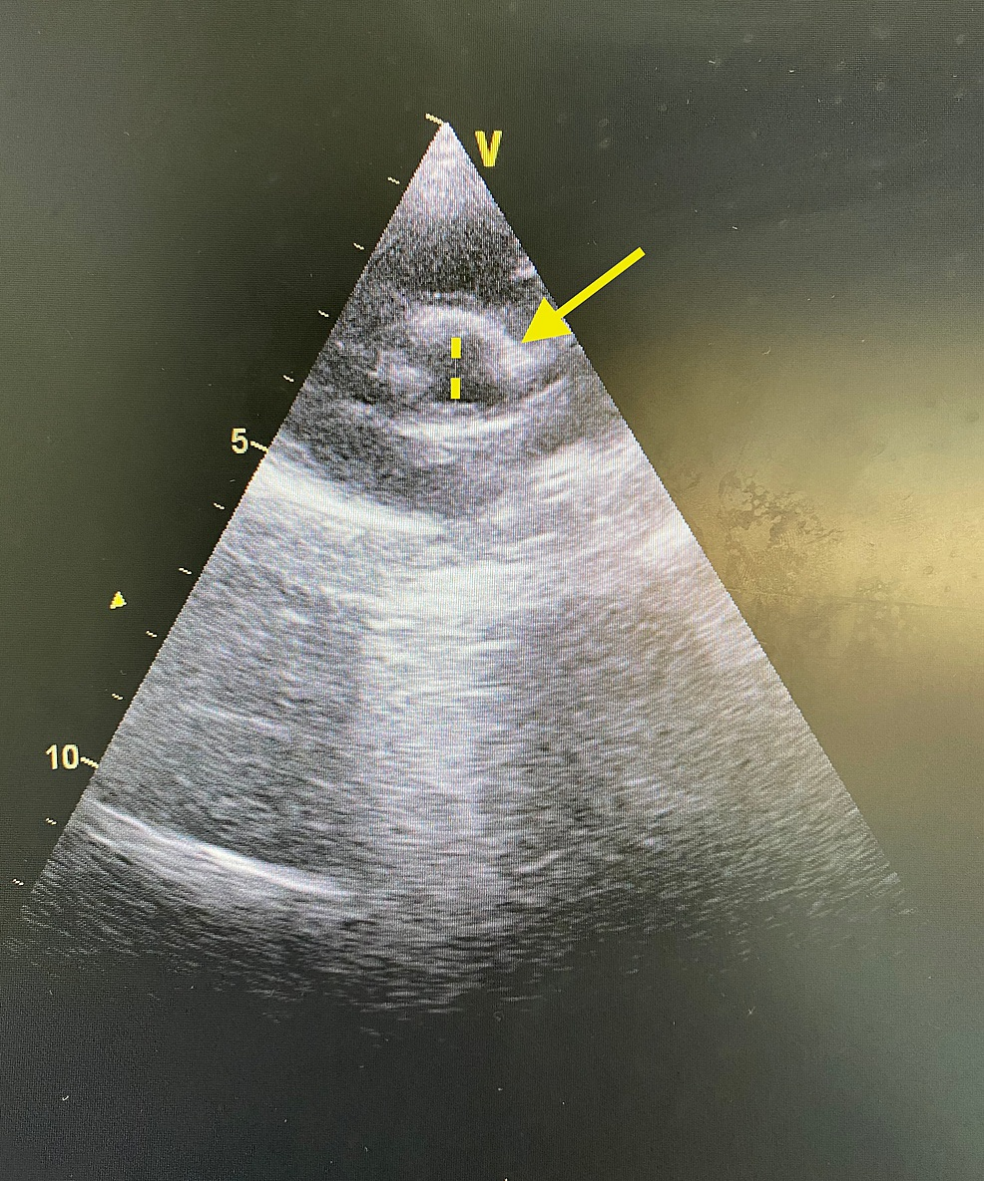
Echocardiogram showing coronary artery prominence with mild dilatation.

A two-month-old Saudi boy, full-term, with no known medical illness, presented with one-day history of high-grade fever and abnormal movements in the form of tonic posture and rolling up of the eyes, with decreased oral intake and activity (Table 1). He was admitted as a case of sepsis to roll out meningitis. Initial examination revealed an irritable febrile infant with unremarkable rest of examination. Initial laboratory tests were significant for leukocytosis and anemia (Table 2). A full septic workup was performed, including a lumbar puncture, and empiric antibiotics were started. After admission, the patient's condition began to improve, and fever decreased on the 4th day. All cultures taken (blood, urine, and cerebrospinal fluid (CSF)) were negative except for pleocytosis in the CSF. On the 7th day, the fever increased again and the family was transferred to another hospital at their request to get another opinion. On the 11th day after the initial fever, the patient was readmitted to our hospital where the fever continued and a generalized rash developed. On examination, he was highly irritable, tachycardic (180 beats/min), febrile (38 °C), tachypneic (56 breaths/min) with wide pulse pressure (96/48) with generalized maculopapular rash and bilateral nonpurulent conjunctivitis with skin peeling on hands and feet. Laboratory findings showed leukocytosis with anemia and hypoalbuminemia. Echocardiogram showed prominent coronary arteries and pericardial effusion (Figure 2). Following these findings, a diagnosis of Kawasaki was made and the associated signs of hypoperfusion led to the diagnosis of Kawasaki shock syndrome. The patient was transferred to the PICU where he received IVIG 2 g/kg and aspirin 80 mg/kg/day every six hours (anti-inflammatory dose). The patient responded well to the IVIG and the fever resolved completely within two days, after which he was switched to the anticoagulant dose of aspirin 5 mg/kg/day. The patient was able to be discharged home after four days, with cardiac follow-up at six weeks, as recommended for patients with Kawasaki disease.

**Table 1:** Clinical features of children with Kawasaki disease shock syndrome.

Clinical feature	Patient 1	Patient 2	Patient 3
Fever	+	+	+
Jaundice	+	-	+
Pruritis	+	-	-
Conjunctivitis	+	-	+
Cyanosis	-	+	-
Angular stomatitis	+	-	+
Lip cracking	+	-	+
Maculopapular rash	+	+	+
Cervical lymphadenopathy	+	-	-
Extremities peeling	-	+	-
Extremities erythema	-	+	-
Anorexia	+	+	+
Emesis	+	-	+
Abdominal pain	-	-	-
Diarrhea	+	-	-
Weight loss	+	-	-
Dehydration	+	-	+
Joint pain	-	-	-
Abnormal movement	-	+	-
History of upper respiratory tract infection (URTI) symptoms	+	-	+

**Table 2:** Laboratory and radiological findings of children with Kawasaki disease shock syndrome.

Laboratory test	Patient 1			Patient 2	Patient 3		
Complete blood count							
Hemoglobin	6.8			8.6		8.9	
	Reference level: 11.1-12.6 mg/dL						
White blood cells	14.48			17.3		11.83	
	Reference level:6-17.5x10^3 ^mg/dL						
Platelets	257			368		304	
	Reference level: 150-350x10^3^						
Blood chemistry tests							
Total bilirubin (TBIL)	11.6			0.49		11.1	
	Reference level: 0.00 to 1.00 mg/dL						
Direct bilirubin (DBIL)	7.2			0.32		10.62	
	Reference level: 0.00 to 0.20 mg/dL						
Albumin	7.2			32.8		16.6	
	Reference level: 34 to 54 g/L						
Aspartate aminotransferase (AST)	76.2			20.7		30	
	Reference Level:15 to 37 IU/L.						
Alanine aminotransferase (ALT)	39			19.2		40	
	Reference level:0.00 to 35 IU/L.						
Inflammatory markers							
Erythrocyte sedimentation rate (ESR)	107			28	107		
	Reference level: 0 and 20 mm/hr						
D-Dimer	3.2			-	-		
	Reference level: less than 0.50						
Coagulation profile							
Prothrombin time (PT)	13.5			-	13.6		
	Reference level: 11 to 13.5 seconds						
Partial thromboplastin time (PTT)	42.2			-	23		
	Reference level: 25 to 35 seconds						
International normalized ratio (INR)	1.19			-	1.03		
	Reference level: 0.8 to 1.1						
Cerebrospinal fluid (CSF) analysis							
White blood cell count		-		8	38		
		Reference level: 0-6					
Glucose level		-		73	63		
		Reference level: 40 to 70 mg/dL					
Protein level		-	40				42
		Reference level: 5 to 40 mg/dL					
Imaging							
Abdominal ultrasound	Unremarkable			-	Liver enlargement		
Chest X-ray	-			Unremarkable	-		
Echocardiogram	Normal			Pericardial effusion with prominent coronary arteries.	Pericardial effusion		

Case 3

**Figure 3:**
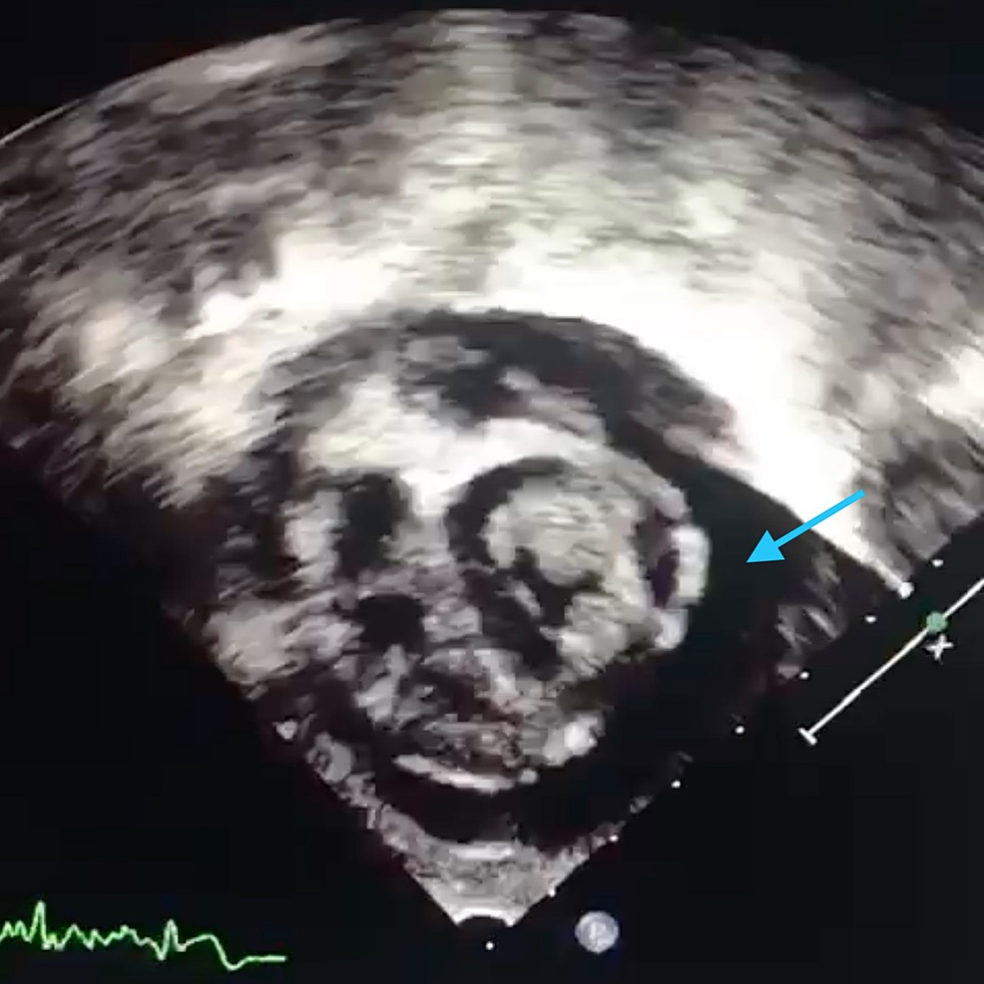
Echocardiogram subcostal short axis at the level of the papillary muscle showing pericardial effusion.

An eight-month-old Saudi boy with glucose-6-phosphate dehydrogenase (G6PD) deficiency presented with a five-day history of fever, jaundice, generalized rash, bilateral nonexudative conjunctivitis with decreased activity, and poor oral intake in the last three days before presentation. Physical examination revealed an irritable infant with depressed sensorium, febrile (39 °C), hypotensive (77/44) with delayed capillary refill time (4 seconds) (Table 1). Initial laboratory tests revealed direct hyperbilirubinemia, anemia, elevated inflammatory marker, elevated liver enzymes, and metabolic acidosis. The patient was rescued with a fluid bolus and a complete septic workup was performed (blood, urine, and CSF) and broad-spectrum antibiotics were started. Echocardiogram showed pericardial effusion with good myocardial contractility (Figure 3). These physical and laboratory findings in conjunction with echocardiography imaging are consistent with incomplete Kawasaki disease and with the presence of hypoperfusion in the form of hypotension, alterations in the sensorium, and hyperbilirubinemia as evidence of impaired conjugation in the liver, leading to the diagnosis of Kawasaki disease shock syndrome. Therefore, the patient was treated with IVIG 2 g/kg and an anti-inflammatory dose of aspirin 80 mg/kg/day divided into four administrations. The patient began to improve on the second day, the fever decreased, and the rash faded. He was switched to an anticoagulant dose of aspirin (5 mg/kg/day) after being fever free for 2 days. The jaundice took another six days to fully improve. At this point, the patient was discharged home on aspirin and referred to cardiology for follow-up in six weeks.

## Discussion

Since the first reported case with KD, the highest incidence of this disease has been in Japan and among the Asian population. Although Saudi Arabia is one of the Asian countries, the exact incidence of KD is not known. However, available data on KD in the Riyadh region, Saudi Arabia show an annual increase in KD cases [4]. According to Dr. Kawasaki, KD occurs more frequently in males and in children under four years of age [2], which was also reflected in this study. The gender dominance is controversial, in Kanegaye et al., KDSS is more common in females as the firstly reported cases, while in Zhang et al., males were in the majority. In our study, males were in majority [3,5].

Many theories attempted to determine the etiology and risk factors of the disease. One of them suggested that an unidentified infection could trigger the immune response that causes KD in genetically suspected individuals [1,2]. This was observed here in the study, and especially in the era of COVID 19 infection, where accurate co-recording and documentation of a previous or current COVID-19 infection increases the association between infections and the triggering of Kawasaki disease or multisystemic inflammatory shock in children with COVID-19 infection (MIS-C) [1,6,7].

Many studies report that more than one-third of KD patients do not meet the classic criteria and are diagnosed as incomplete KD [8]. Patients with incomplete KD tend to be diagnosed later than those with the classic presentation. These patients have at least five days of fever and fewer than four criteria in addition to laboratory or echocardiographic findings suggestive of KD [1,3,8].

KDSS is defined as the concomitant presence of Kawasaki disease with accompanying signs of hemodynamic instability [3]. The cause of hypotension has not been specifically determined. However, it is thought to be caused by capillary leakage and cytokine dysregulation as a complication of vascular inflammation of KD [1,3]. All patients in the present series met the inclusion criteria of KDSS with the diagnostic criteria of KD. Compared with multisystemic inflammatory shock in children with COVID 19 infection, the patients were significantly more hemodynamically unstable and had a higher incidence rate. Cardiovascular collapse was the cardinal sign in both KDSS and MIS-C. Globally, myocardial and valvular dysfunction are observed, but compared to what was observed here and in Almoosa et al. peripheral vascular dysfunction is the main feature with minimal myocardial impairment [1,6,7]. 

Gastrointestinal manifestations in KDSS are observed due to hepatic dysfunction such as cholestasis and hepatic synthetic dysfunction leading to coagulopathy as observed here in one case. The exact mechanism of this presentation is unknown. It has been hypothesized that vascular inflammation of KD spreads to the liver and gallbladder or lymph node enlargement causes obstructive jaundice [9]. The extent of coagulopathy is much more severe in MIS-C compared to KDSS. Vascular leak syndrome is the main hypothesis for the presenting hypoalbuminemia [1,3,9].

Central nervous system involvement in KD is not uncommon, with a wide spectrum of involvement. Aseptic meningitis was observed here with a higher percentage than previously reported, as CSF analysis showed pleocytosis with negative culture in two patients (66%). This might be related to capillary leak syndrome associated with KDSS [3,10].

Respiratory failure and mechanical ventilatory support did not occur in KDSS. On the other hand, pneumonia or respiratory failure may occur in MIS-C, which may require mechanical ventilatory support or oxygen supplementation [3,7-10].

Treatment of KDDS is mainly based on the use of immunomodulatory and immunosuppressive agents. Intravenous immunoglobulin (IVIG) seems to be the agent of choice. Its mechanism of action is controversial, but recent studies suggest that the anti-inflammatory effect of IVIG is mediated by up-regulation of T-regulatory cells and downregulation of Th17 signaling pathways. IVIG was able to shorten the duration of the disease and prevent coronary artery aneurysm (CAA). Therefore, early detection and treatment of KD are crucial to achieve the best outcomes. The American Heart Association (AHA) recommends starting IVIG treatment within 10 days of disease onset, ideally within seven days. If diagnosis is delayed beyond 10 days, treatment should only be started with fever or aneurysms with laboratory evidence of active inflammation are present [1,3,10,11]. This applied to the patients in this study, even at late presentation of the second case, there was persistent fever and evidence of early coronary artery changes by echocardiography.

KDSS are at higher risk for coronary artery dilatation and are more likely to require an additional dose of IVIG. This observation contrasts with our first and third patients, both of whom responded well to a single dose of IVIG and did not develop coronary complications, which may contribute to early treatment and detection of KDSS.

In addition, KD patients who have neurological manifestations have a relatively increased risk of IVIG resistance [12]. Encouragingly, patients who had aseptic meningitis responded well to treatment, although there was a late presentation in the second case.

Corticosteroids may have a role in resistance cases to IVIG or in combination with IVIG when CAA are large and have a poor outcome. It is used with high doses (pulse therapy) and has the ability to change the size of the CAA. However, in very resistant cases, tumor necrotic factors such as infliximab can be used, but unfortunately, this will not change the outcome of CAL [1,13].

Aspirin is rarely used in pediatrics because of the risk of Reye's syndrome, but this is not the case in KD. Aspirin has an anti-inflammatory effect by using high doses until suppression of fever, then an anti-platelet role until resolution of CAL [1,13]. In the current study, all patients received both IVIG and aspirin; however, in the first case, aspirin was post-dosed until the coagulation profile normalized.

## Conclusions

KD, a vasculitis of the medium-sized vessels, usually has a benign course if recognized and treated early. A life-threatening event leading to circulatory collapse, commonly referred to as "shock" or in technical terms as "Kawasaki disease toxic shock syndrome," can result, although it is rare. The signs and symptoms of KDSS can be vague and confusing, but one should always be suspicious, especially if fever persists. Immunomodulatory agents are the drug of choice to control the disease and prevent the adverse sequelae, especially coronary artery aneurysms. Finally, fever is the most presenting symptom in children with a wide range of differential, vasculitis of such and if you do not think about it, you will miss it.

## References

[1] Son MBF, Newburger JW (2018). Kawasaki disease. Pediatr Rev.

[2] Kawasaki T (1967). Acute febrile mucocutaneous syndrome with lymphoid involvement with specific desquamation of the fingers and toes in children. Arerugi.

[3] Kanegaye JT, Wilder MS, Molkara D (2009). Recognition of a Kawasaki disease shock syndrome. Pediatrics.

[4] Burgner D, Harnden A (2005). Kawasaki disease: what is the epidemiology telling us about the etiology?. Int J Infect Dis.

[5] Zhang MM, Shi L, Li XH, Lin Y, Liu Y (2017). Clinical analysis of Kawasaki disease shock syndrome. Chin Med J.

[6] Almoosa ZA, Al Ameer HH, AlKadhem SM, Busaleh F, AlMuhanna FA, Kattih O (2020). Multisystem inflammatory syndrome in children, the real disease of COVID-19 in pediatrics - a multicenter case series from Al-Ahsa, Saudi Arabia. Cureus.

[7] Dufort EM, Koumans EH, Chow EJ (2020). Multisystem inflammatory syndrome in children in New York state. N Engl J Med.

[8] Yu JJ (2012). Diagnosis of incomplete Kawasaki disease. Korean J Pediatr.

[9] Feldstein LR, Rose EB, Horwitz SM (2020). Multisystem inflammatory syndrome in U.S. children and adolescents. N Engl J Med.

[10] Chen PS, Chi H, Huang FY, Peng CC, Chen MR, Chiu NC (2015). Clinical manifestations of Kawasaki disease shock syndrome: a case-control study. J Microbiol Immunol Infect.

[11] McCrindle BW, Rowley AH, Newburger JW (2017). Diagnosis, treatment, and long-term management of Kawasaki disease: a scientific statement for health professionals from the American Heart Association. Circulation.

[12] Liu X, Zhou K, Hua Y (2020). Neurological involvement in Kawasaki disease: A retrospective study. Pediatr Rheumatol.

[13] Dhanrajani A, Yeung RSM (2017). Revisiting the role of steroids and aspirin in the management of acute Kawasaki disease. Curr Opin Rheumatol.

